# A215 CAUSE-SPECIFIC MORTALITY IN PATIENTS WITH CIRRHOSIS: A POPULATION-BASED COHORT STUDY

**DOI:** 10.1093/jcag/gwab049.214

**Published:** 2022-02-21

**Authors:** P Wang, M Djerboua, J Flemming

**Affiliations:** Queen’s University, Kingston, ON, Canada

## Abstract

**Background:**

Cirrhosis is a major global health concern with increasing mortality secondary to cirrhosis and chronic liver diseases observed both in the United States and Canada. However, population-level causes of death among patients with cirrhosis in North America have not been reported.

**Aims:**

The aim of this study was to describe cause-specific mortality in patients with cirrhosis stratified by cirrhosis etiology.

**Methods:**

Retrospective cohort study using linked administrative healthcare data from Ontario, Canada. Adult patients with cirrhosis 2000/01/01-2017/12/31 were identified and etiology of liver disease was assigned as hepatitis C (HCV), hepatitis B (HBV), alcohol-associated liver disease (ALD), non-alcoholic fatty liver disease (NAFLD), or autoimmune liver disease (AI)/other using validated algorithms for cirrhosis (sensitivity [sn] 99%, specificity [sp] 79%) and cirrhosis etiology (sn 75%-97%, sp 95%-100%). Patients were followed until death, liver transplant, or end of study. The *primary outcome* of cause of death was defined based on the top 10 causes of death reported by Statistics Canada; however, hepatocellular carcinoma and cholangiocarcinoma were included in liver-related deaths as opposed to malignant neoplasms. The proportion of deaths by each cause was described, stratified by cirrhosis etiology. The cumulative incidence of death with liver transplant and hepatocellular carcinoma as competing risks were calculated at 1, 5 and 10 years.

**Results:**

202,022 patients with cirrhosis were identified (60% male sex, median age 56 years [IQR 46–67], 52% NAFLD, 26% ALD, 11% HCV, 5% HBV, 6% AI/Other). Overall, 81,428 (40%) patients died after a median follow-up of 5 years (IQR 2–12) and 3,024 (2%) patients received liver transplant. The overall leading cause of death was liver-related (32%) but varied substantially by cirrhosis etiology. Liver-related deaths were highest among those with viral hepatitis (HBV 56%, HCV 52%), and lowest in NAFLD (20%). In NAFLD cirrhosis, the most common causes of death were non-hepatic malignancy (26%), followed by a composite of cardiovascular disease, cerebrovascular disease, or diabetes (22%).

**Conclusions:**

Although the overall leading cause of death in patients with cirrhosis is liver-related, the most common causes of mortality in patients with NAFLD cirrhosis is non-hepatic malignancy, cerebrovascular disease, and diabetes. This supports the involvement of multidisciplinary teams and healthcare providers for patients with NAFLD cirrhosis to optimize appropriate cancer screening and management of co-morbid cardiovascular and metabolic conditions.

**Funding Agencies:**

None

